# The Gallinger Bill

**Published:** 1898-03

**Authors:** 


THE GALLINGER BILL.—There are grave reasons to fear that this bill, ostensibly in the interests of humanity, and to prevent cruelty to animals, will become a law, and unless sufficient influence with Senators is brought to bear to defeat it. Every friend of science, who appreciates the benefit of experimental research in animal physiology should appeal at once to his representatives at Washington, making them acquainted with the true animus of the attempt. Senator Gallinger is a homeopathic doctor, and the true object of this nefarious measure is to prevent laboratory experiments on frogs, turtles, snakes, guinea-pigs and rabbits. The Loud bill would have passed had not the publishers all over the United States taken active steps to defeat it by explaining to Congressmen what it meant.

